# Supplementary figures and images for: Are haloclines distributional barriers in anchialine ecosystems? Physiological response of cave shrimps to salinity
Source: PLoS One. 2024 Jul 25;19(7):e0305909. doi: 10.1371/journal.pone.0305909 (PMC11271914; doi:10.1371/journal.pone.0305909)

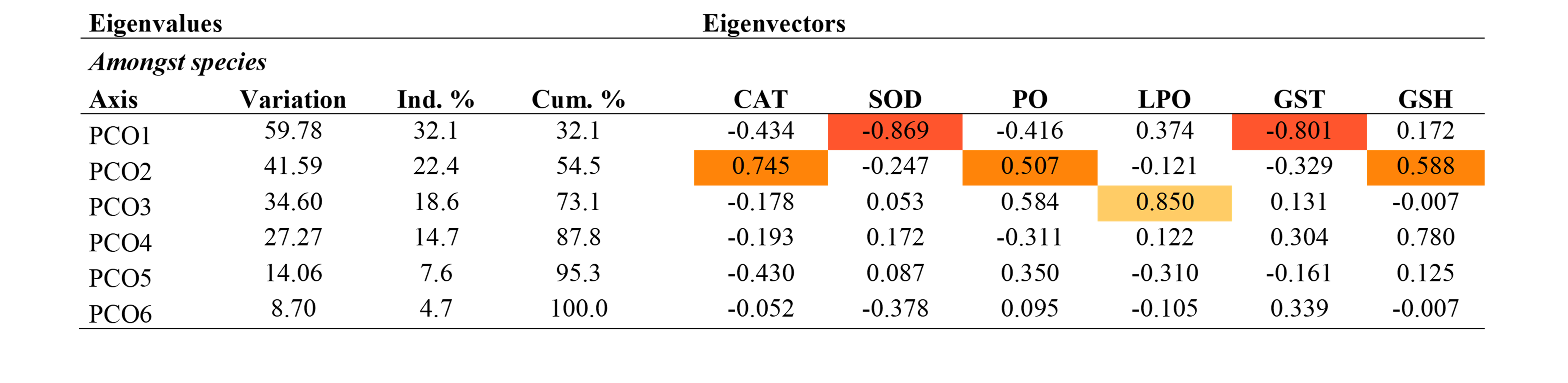

Supplement: S1 Table — The amount of explained variation by each principal coordinate is expressed in absolute, relative and cumulative magnitudes. Antioxidative enzymes were catalase (CAT), glutathione S-transferase (GST), total glutathione (GSH), and superoxide dismutase (SOD). Oxidative damage was quantified through protein carbonylation (PO) and lipid peroxidation (LPO). (TIF) [file pone.0305909.s002.tif]

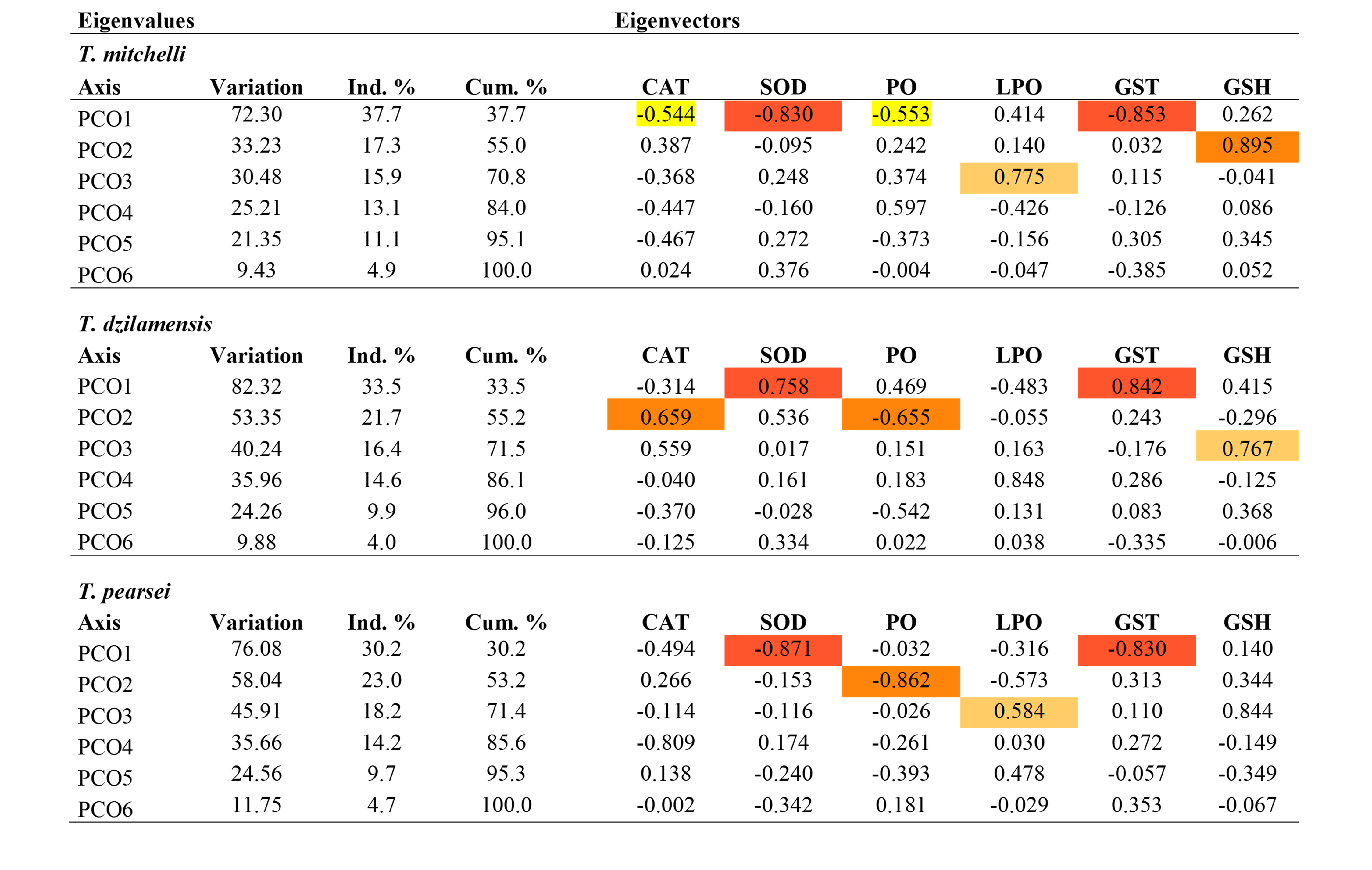

Supplement: S2 Table — The amount of explained variation by each principal coordinate is expressed in absolute, relative and cumulative magnitudes. Antioxidative enzymes were catalase (CAT), glutathione S-transferase (GST), total glutathione (GSH), and superoxide dismutase (SOD). Oxidative damage was quantified through protein carbonylation (PO) and lipid peroxidation (LPO). (TIF) [file pone.0305909.s003.tif]

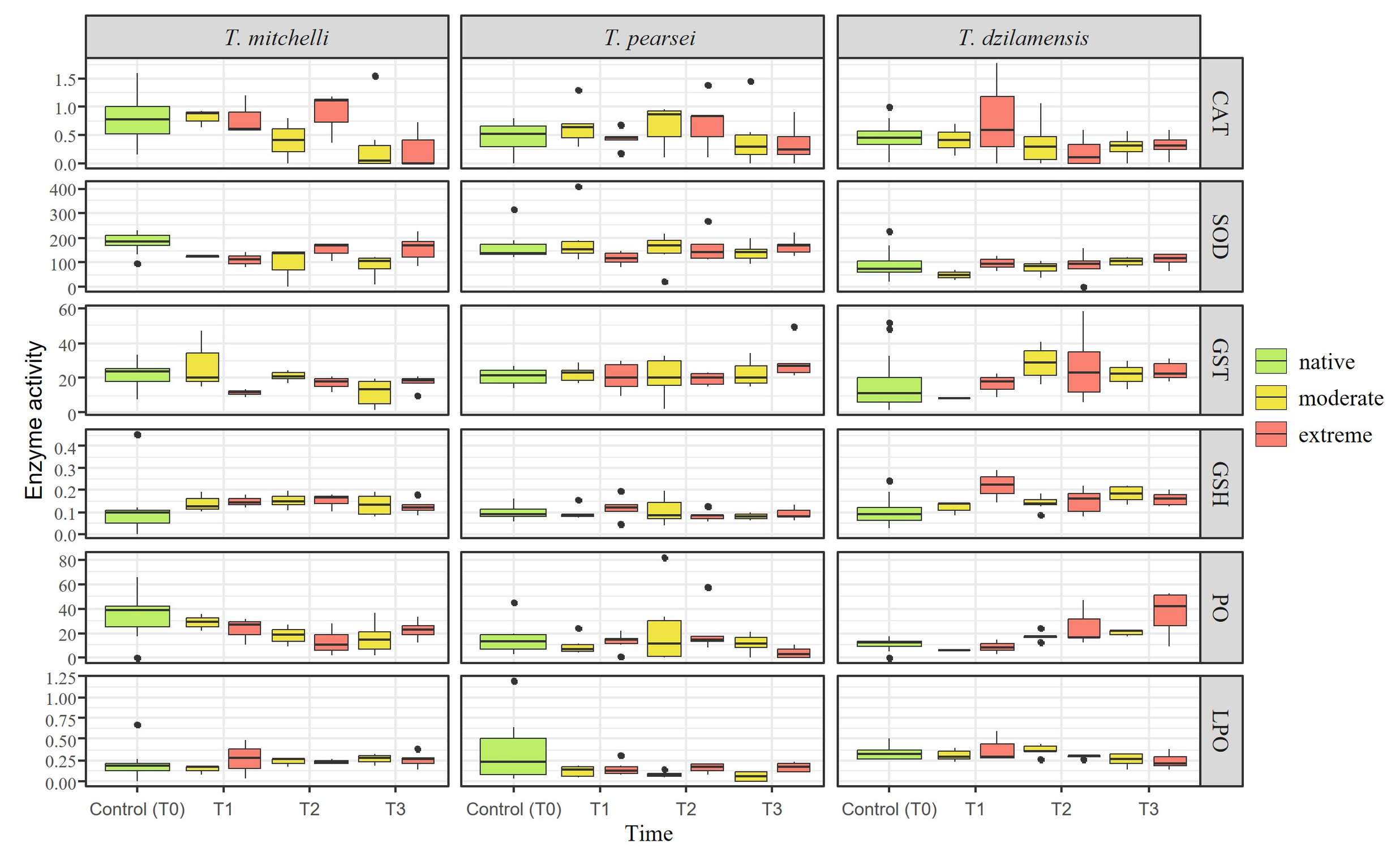

Supplement: S1 Fig — (TIF) [file pone.0305909.s004.tif]
