# Supplementary material for: Are haloclines distributional barriers in anchialine ecosystems? Physiological response of cave shrimps to salinity
Source: PLoS One. 2024 Jul 25;19(7):e0305909. doi: 10.1371/journal.pone.0305909 (PMC11271914; doi:10.1371/journal.pone.0305909)
Supplement: Attachment — Submitted filename: Response to reviewers.pdf [file pone.0305909.s005.pdf]

Response to reviewers
Research article PONE-D-23-27494

Are haloclines distributional barriers in anchialine ecosystems? Physiological response of cave shrimps to salinity.

Reviewer #1:

1) More detail needs to be given about acclimation times during the metabolic rate experiments. After shrimp were collected from the habitats and brought up, how long were they allowed to adjust to their new settings before metabolic rate measurements began at the different salinities? In most experiments of this kind, animals are allowed to settle in for a period of time before measurements begin (or before the data are recorded), because handling stress can cause MR to increase. In all the species, MRs are high initially at test salinities and then decrease over time. The authors interpret this as a physiological response to salinity, but it may be a result of handling stress during setting up the experiment and/or transfer of animals to the new salinity. If shrimp were brought up and then placed in the native salinity for control measurements over 150 minutes, the authors could address this by showing the same time series data (over 150 mins) for the control vs. test salinities to see if MRs were initially elevated there as well before lowering. Based on Fig 1, it seems this might not be the case, so the authors may not be able to disentangle time from salinity. In any case, stress due to handling and initial responses to the experimental apparatus (not salinity) should be discussed. The samples sizes for each measurement/experiment and species should also be referenced in the main text, not just Fig 1.

Answer. We have expanded the methods section, describing the time elapsed from collection to experimentation, added the number of individuals used for each trial and a note stating that none were used more than once in any trial. We have also explained how we calculated the RMR in native conditions and added a phrase explaining why this was used as a reference for all other trials. Figure 2 has been modified and now reads: control (0-150 min).

The objective of these experiments was to characterize the physiological response of wild individuals in situ, without any acclimation to the confinement or more traditional experimental setups. Because we observed droppings and depletion of the digestive tract soon after *Typhlatyas* had been collected, we strived to begin the experiments as quickly as possible, to avoid experimenting with “hungry” individuals.

Finally, handling stress was similar for individuals in all RMR trials, allowing for valid comparisons of the effect of the salinity on all indicators through time both in native (control) and challenging salinity scenarios. In particular, the RMR of control individuals was assumed to be constant throughout the 150 minutes, so any changes observed after being transferred to challenging scenarios could be attributed to salinity.

2) In interpreting the results, the authors should consider direction of salinity challenge as a possible explanation. T. mitchelli and T. pearsei were undergoing freshwater to seawater transfers and had increased stress/low survival. T. dzil. underwent an opposite transfer and had high survivorship. Given that the atyids are a freshwater family, increased salinity might be a natural stressor, while T. dzil. has switched to being a seawater specialist, but still maintains the ability to survive in freshwater. In truly euryhaline species, researchers often acclimate all species to the same salinity and then undergo a transfer in the same direction. While that isn't possible in this system, it is worth noting that direction of transfer also covaried with tolerance here. It should also be noted that because of the nature of the system, an entirely controlled and comparable experiment is not possible.

Answer. We have added a paragraph to the discussion addressing the matter, emphasizing the freshwater nature of the atyids, the last common ancestor of the Yucatan-Cuba clade and how the difference of *T. dzilamensis* effectively colonizing the MG environment is not shared by its sister species.

3) I found the presentation of the results somewhat confusing. This is especially true for figures 3 and 5. For fig 3 the main issue is that the PCA is difficult to interpret given the overlap of the points in many panels. Adding a mean and ellipse for each treatment would go a long way towards letting the reader see how the treatments are affecting the PCA values. I'd also suggest adding a set of supplementary figures similar to Fig2 and 4 for each of the enzymes examined, so readers interested in a particular enzyme can see how its activity changed with salinity in each species. Similarly, in Fig 5 the interesting comparison is how AS changes with salinity within each species, not differences between species at a particular salinity. I'd suggest reformatting this figure to match the layout in Figs. 2 and 4.

Answer. We insist on keeping Fig. 3 without adding ellipses. We believe this figure already displays two different responses: impacts regarding the magnitude of the salinity change (triangles and squares), and impacts derived by the time they are maintained at this new salinity (tones of blue). Furthermore, we show the enzymes profile in native conditions (green) without any salinity changes. We agree that this figure can be challenging because it condenses a lot of information. However, taking proper time to analyze this figure is important to understand how the changes in time and at different salinities have different impacts among these species and treatments. In hope of being clearer we have extended the description of these results and included a supplementary figure (SF1), where enzymes are shown for each species over time and salinity treatment, as suggested.

We had originally submitted fig 5 in this manner for consistency (matching the layout) with figs 2 and 4, and because the comparison within each species was simple. However, we agree with the suggestion of changing the order in fig 5.

4) Minor comments

1) Line 23 – "by the haloclines" should be "by haloclines"

Answer. Modified.

2) Line 32 – "novel physiological characteristics" is too vague. I suggest removing this and emphasizing that some species may be euryhaline while others are restricted to the freshwater portion of the habitat.

Answer. Modified.

3) Line 52 – remove “the” before “rapidly advancing climate change”

Answer. Removed.

4) Line 58 – change “is” to “are” and remove “the”

Answer. This has been modified and now reads “Acute changes in salinity are one of the defining characteristics of anchialine environments”

5) Line 113 – change “resource” to “resort”

Answer. Modified.

6) Line 136 – list collection parameters for T. pearsei here as well.

Answer. This has been modified and added to the Animal Collection subsection in Methods.

7) Line 190 – change “trough” to “through”

Answer. Modified

8) Line 225- change “essay” to “assay”

Answer. Modified

9) Line 309 – what statistics were used to analyze the aerobic scope data? I didn’t see this in the statistics section.

Answer. A new paragraph has been added explaining why only descriptive statistics were used to analyse the aerobic scope.

10) Line 319 – the laying out of all the different comparisons is quite confusing. I might suggest condensing this section and having a few lines for each species stating the general trend and then maybe the significant differences.

Answer. We have re-written the results section in order to articulate RMR values that were measured with the results of statistical procedures, and the observed behaviours of *Typhlatya* throughout trials.

11) Prior to getting into the MR data, I’d suggest a few lines outlining the behavior and survival of each species instead of listing this later. This will give the reader an idea of general stress responses before getting into the MR data. In general, I think its ok to have a paragraph for each species where the behavior/survival observations are noted first, then the MR trends and significant differences. It is also difficult to keep track of the species if one isn’t familiar with them, so it is useful to reiterate for each species where they were collected (FW or SW) and which direction they were transferred in.

Answer. We have restructured the results section and hope these are more straightforward.

12) Line 563 – this is a good point that I would like to see expanded upon – increased MR could be interpreted as an adaptive/beneficial response (they are able to mount a physiological response) or a maladaptive response (they are burning lots of energy). Because these interpretations are polar oppsites, its important to interpret the data here in both ways and discuss what either interpretation could mean for stress, etc.

Answer. We have changed the structure of the discussion and have included a paragraph in lines 643 – 676 which addresses this point.

13) Line 615: “which can”?

Answer. Deleted.

Reviewer #2:

Dear authors! I have reviewed your manuscript: “Are haloclines distributional barriers in anchialine ecosystems? Physiological response of cave shrimps to salinity.” It is a relevant and timely study bringing novel data that highlight potential issues certain species might experience in the rapidly changing world due to changes in salinity in a sensitive and rare environment. I did not find any major issues regarding the content but think that the paper is poorly organised; not clearly written and needs a major makeover of many parts. Please see my comments and suggestion below on how to improve this.

INTRODUCTION

1) I think the introduction needs a thorough makeover. A substantial part of text (3rd, 4th, and 5th paragraph) is now very technical, devoted to introducing common, well-known physiological traits, their proxies and how to measure these. This has nothing to do with the story you are telling but is a much more related to the methods you used to assess sensitivity to salinity. I recommend you move most of this text to the appropriate parts of the methods section. In the intro, possibly just in the last paragraph, mention just the essentials. This will free-up space which I suggest you use to build a more solid story. I think you should make climate change (and anthropic) driven changes in environment and their effect on species living along haloclines and potential conservation issues as your main thread and strengthen it.

Answer. We have restructured the introduction, moving most of the suggested paragraphs into a new the methods section (“Methodological background”), and we have modified our introduction to respond to environmental changes and their risk under current climate change projections.

2) Please include relevant recent review studies on threats to cave life and habitats like Mammola (many papers), Wynne et al. 2021, but also one of the landmark papers on anchialine cave ecology (Sket, 1996). Also, more specifically introduce what is known of salinity sensitivity/tolerance in cave animals in general. Due to increased threats of anthropic salination of freshwater, recently a few relevant studies were published (e.g., Castaño-Sánchez et al 2020, Jemec et al. 2022) and I suggest you refer to them either in introduction or devote a paragraph for this in discussion. Finally, please improve the presentation of your model system in the 6th paragraph. Say why are these shrimps a good model for your question, and let the reader know the essentials of the model system required to understand this. Sket, B 1996: https://doi.org/10.1016/0169-5347(96)20031-X; Mammola et al: https://doi.org/10.1111/brv.12642; https://doi.org/10.1111/brv.12851; https://doi.org/10.1093/biosci/biz064; https://doi.org/10.1177/2053019619851594; Wynne et al. 2021: https://doi.org/10.1111/conl.12834; Castaño-Sánchez et al 2020: doi: 10.1038/s41598-020-69050-7; Jemec et al. 2022: https://doi.org/10.1016/j.ecoenv.2022.113456

Answer. We have included the suggested references in the introduction, and have devoted a paragraph in the discussion to salinity tolerance, as suggested. Additionally, we expanded the description of why we chose to work with Typhlatya species from Yucatan.

3) First lines: support your claims with references.

Answer. Modified.

4) Lines 54-57: first paragraph of the intro should introduce the broad topic; this interrupts the flow, please remove and reuse maybe in the last parts of the introduction; for example the 6th paragraph

Answer. We have re-structured the introduction as suggested and hope this has a better flow.

5) Lines 56-57: No need for abbreviations, in my opinion. Just use the full phrasing throughout the manuscript.

Answer. We insist on keeping this abbreviation in order to lighten the reading and avoid an excessively wordy manuscript.

6) Lines 58-60: flip the order of the sentence

Answer. The phrase has been modified.

7) Line 63: FG and MG, usually your abbreviate as FGW and MGW.

Answer. This has been addressed throughout the manuscript.

8) Lines 68-69: impossible to understand without checking Box 1, but actually also not explained in the box, but in the 3rd paragraph of intro. Please rewrite so that the question is instantly understandable.

Answer. The glossary box now includes the proper terms and the phrase has been modified so that reader does not need to refer to the box.

9) Lines 71: no need for a capital letter in Oxygen

Answer. Modified.

10) Lines 78: a fullstop is missing after references

Answer. Modified.

11) Lines 85: start a new sentence after “decreases”

Answer. Modified.

12) Lines 86: rephrase “have exceeded”

Answer. This sentence has been modified and now reads “Negative AS values are only possible for short periods of time, as this implies that life maintenance costs exceed the ATP production capacity.”

13) Lines 90 & 95: lethal in italic

Answer. Modified.

14) Line 99: comma after demand

Answer. Modified.

15) Line 105: no need to use capital letters.

Answer. Modified.

16) Line 121: Please reconsider expressing your units of salinity (UPS) in the new standard, TEOS-10, so in promilles, g/kg, also dimensionless.

Answer. We have changed the PSU to the standard TEOS-10 for salinities from 2-35 and IES-80 for salinities under 2 PSU. We have stated the absolute salinity in the salinity scenarios and continued with the Ps (practical salinity) throughout the manuscript as a way to simplify the numbers for the readers.

17) Lines 121-122: delete the sentence. It only adds confusion. Not clear what is this Yucatan-Cuba clade. Are Typhlatya part of it or not.

Answer. This has been modified and moved to the discussion section, as this information gives an evolutionary background to the *Typhlatya* species we use in this work. The sentence now reads: In particular, the *Typhlatya* pertaining to the Yucatan + Cuba clade were found to have a low salinity most recent common ancestor.

18) Lines 124: not clear what SGW is. Did you mean MGW?

Answer. Yes, thank you, this has been modified and now reads MG.

19) Lines 126-130: very long sentence and thus hard to comprehend. Consider breaking into two or three shorter sentences.

Answer. Sentence has been divided.

20) Lines 131: and should not be written in italic

Answer. Modified.

21) Lines132: not clear what you mean by "compared such consequences". Rephrase.

Answer. We have modified to "compared such responses through…"

22) Line132: change metabolic rate to routine metabolic rate, otherwise the abbreviation does not match

Answer. Modified.

METHODS

Animal collection

1) It is not clear where T.pearsei was collected: in the solely freshwater cenotes? It is also not clear whether T. michelli was collected also from these two, or just from the Pandrosa system. I guess T.dzilamensis was collected only in the last system. From the next subchapter it seems you did not sample in the Tza Itza cenote at all. Why do you mention it here?

Answer. We have added the following sentence ". *T. pearsei* were collected from the cenote pool of the exclusively freshwater cenote Nohmozon at night." And we have further explained that T mitchelli was collected from both Tza Itza and Ponderosa for the control individuals only.

2) Already in the introduction, you could say and explain more about the distribution of these shrimps and if and how often they co-occur. This relates to my previous comment in intro on improving model system description. Also, I suggest you add a sentence to say how far apart are these cenotes, are they connected somehow, and so on. So improve the description of geography, collecting sites. You should say the total number of animals per species. Was it time consuming to catch them? Are these numbers a significant proportion of the populations? Add this info so the reader can get a feeling for the effort it takes just to collect animals in this difficult habitat, and can better appreciate your work.

Answer. We have added a second paragraph to the *Animal collection* subsection which now describes with more detail how the individuals were collected and the effort invested to obtain the biological samples. We have also added a description of where and how far from the coast these cenotes are located.

3) Line 139: capital letter for system

Answer. Modified.

4) After animal collection you could open a new subchapter in which you could explain the choice of your physiological traits and proxies, that I suggested to move from the introduction into the methods. Next subchapter would be "experimental setup" where you describe the materials you used in your experimental protocols. Now this is included mostly in Experiment 1.

Answer. We have briefly explained the concepts required to understand each of the methods used in our study in a new section ("Methodological background") helps separate this important information from the Introduction at the same time it provides clarity as to why and how these methods are a means to reach our objectives.. We have also included a general description of the

rationale underlying all the experiments: compare physiological and biochemical indicators of individuals of the 3 species exposed to a native and challenging salinities.

Salinity scenarios

5) Please explain how you measured salinity at collection sites. Did you measure also throughout the vertical column to reconstruct the halocline?

Answer. The leader and first diver in the cave diving team carried a Hydrolab DS5 to ensure the column would be measured without disturbance. Results of these measures have been published in Chavez Solís et al 2020, which is why we are not presenting them here again. Additionally, salinity from the collected groundwater was measured with a refractometer prior to any trials. We have added the relevant information in the last paragraph of the salinity scenarios subheading of the methods section.

6) Line 150: a space is missing before the references.

Answer. Modified.

7) Line 158-159: change UPS to PSU

Answer. Modified.

Experiment 1

8) Altogether not clearly described what was done. Very hard to follow. Exclude the experimental setup and focus on the protocol. You should clearly say the sample sizes per species for this part of the experiment and also the subsequent ones. This is not clear enough. Also, not clear whether between the three experiments individuals were reused or a specific individual was used only in one experiment. Please explain.

Answer. We have deeply modified the complete section hoping it is easier to follow. We have added a paragraph addressing the number of individuals collected and used for each of the respirometry trials. We focused on describing the experimental setup and how the entire procedure was carried out.

9) Line 172: change MR to RMR

Answer. Modified.

10) Line 180: change metabolic rate (MR) to routine metabolic rate (RMR)

Answer. Modified.

11) Line 186 & 188: replace weight by mass, these are two different physical quantities and you measured mass, not weight.

Answer. Replaced.

12) Line 189 & 190 & 216 & 235: add space between number and related unit.

Answer. Modified.

13) Line 205: replace Nitrogen by nitrogen.

Answer. Replaced.

Experiment 3

14) It is not clear how the control treatment was performed. Please add a short explanation. I guess you had to weight individuals also for the previous two experiments, but you do not mention it. Add.

Answer. We have added the description of weighing the individuals to the other experiments and have described the control treatments.

Statistical analyses

15) Delete the first sentence and move it to the very end of statistical methods. Say that plots and analyses were done in R. The latter is missing now. Please also include and cite the R packages used as well as functions with which you performed the main model and post-hoc tests.

Answer. We have included a general description of the statistical approach used to analyse the data. We have correctly cited packages and software used to conduct all statistical analyses, both formal hypotheses testing and graphical visualisations.

16) Either add subtitles or start each paragraph so, that it is instantly clear to which of the above experiments and data the analyses relate. Now it is not.

Answer. We have added subtitles indicating which set of data/experiment were the statistical procedures applied to .

17) First paragraph (lines 266-279): I suggest reorganisation of text to improve clarity. First say that you tested for differences in RMR at native salinity between species and how. Second describe testing differences between salinity challenge and time. When describing the permutation ANOVA model please first say what was the dependent variable and what were independent variables, only then move to specifics of the permutational ANOVA. For general understanding it is more important to say you performed a two-way ANOVA model. For factor time use categories in min instead of t1, t2, t3. Lastly, describe how you performed post-hoc tests. Use main effects instead of model terms. You should adjust your p-values for multiple comparisons via one of the many available methods. Did you do it and just forgot to mention it? Please explain in text why you had to use permutational ANOVA and not normal ANOVA. Why did you not include an interaction between the two factors in your models? Also, did you consider performing one permutation ANOVA with an additional third factor “species”? Your models do not allow testing difference between species, which would be the most interesting thing to do in my opinion. The same questions and suggestions apply also to similar model procedures you describe next.

Answer: We chose a permutational approach to analyse the univariate RMR data because both response variables failed to conform to a normal distribution, and because data with strong covariation patterns are rarely expected to comply to multivariate normality. Using a traditional ANOVA (or MANOVA) relies on the theoretical distribution of the *F* statistic, which in turn requires normality in the response variable(s). Permutations of the labels (codes), the raw data, and the residuals under the reduced model is a standard procedure in multivariate contexts, but can be easily applied to the univariate case (i.e. the Fisher exact test). The procedure is based on generating an empirical *F*-distribution under the null hypothesis and assessing the cumulative frequency of values as large as or greater than the observed *F*-value (called pseudo-*F*). The permutational tests using the *t*-statistic follow the same rationale; consequently, permutational p-values obtained in multiple comparisons can be considered exact.

In order to examine whether RMR varied through time (t1, t2, t3 and t4) and if this variation was similar irrespective of the magnitude of the salinity challenge (moderate and extreme), we used a two-factor ANOVA model (time + salinity challenge + interaction). If the interaction term was significant, then changes in RMR through time were different depending on the magnitude of the salinity challenge. This was only true in the case of *T. mitchelli* (see Table 1 in the original

version), but further comparisons of group means for factor time were performed whenever this source of variation was found to be significant.

Because the time span of the response to salinity amongst the three *Typhlatya* was not comparable and moments in time (t1, t2, t3 and t4) differed between species (see Lines 224 - 226 and Figure 1), ANOVAs were applied to each species separately. For the same reason, we only compared the RMR, lactate and the six BI between *Typhlatya* species under their respective native salinities, and used one-factor ANOVA models (species) to do so. We have reorganised the text in the attempt of clarifying the points raised by Reviewer #2, which we found both accurate and thorough. We have also added information as to which are response (dependent) and explanatory (independent) variables in all cases.

18) Second paragraph (lines 280-287): 1) Describe the procedure for baseline (control) data. 2) Describe the procedure for treatment data per species.

Answer: We believe that the way in which controls were statistically compared to salinity treatments is now more clearly explained.

19) Line 280: usually abbreviated as PCA not PCoA.

Answer: A Principal Coordinate Analysis (PCoA) differs from a Principal Component Analysis (usually abbreviated as PCA) in that the former performs an eigen analysis on a resemble matrix of the distance (in this case Euclidian) calculated between all n observations (PCA performs an eigen analysis on the variance-covariance or correlation matrix between all p variables). As a PCA, a PCoA also allows to position objects in a space of reduced dimensionality while preserving their distance relationships as well as possible. Whilst PCA preserves the Euclidean distance between objects, PCoA can be applied to resemble matrices using a variety of distance measures or dissimilarity indices (Legendre & Legendre 1998. Numerical Ecology. 2nd Ed. Chapter 9). PCoA is a recommended procedure in order to obtain the centroids (multidimensional means) and calculate the corresponding SS and MS (Anderson 2017. Permutational Multivariate Analysis of Variance (PERMANOVA) in Wiley Stats Ref: Statistics Reference Online). We applied a Principal Coordinate Analysis to the 6 indicators of the antioxidant system and oxidative cellular damage, and believe it should be abbreviated as PCoA to distinguish it from a PCA.

20) Third paragraph (lines 288-301): It is not instantly clear the here you describe the analyses of PCA scores obtained from data on biochemical indicators. Please improve the text with this in mind. If you join this paragraph with the previous one, would already be helpful. Also, and critical, the MANOVA is insufficiently described. What were the dependent variables? How many PCA dimensions/axes did you include? Again, first describe the analyses of baseline data, controls, then move on.

Answer: As explained before, we did not use PCA or a standard multivariate analysis of variance (MANOVA) to analyse the biochemical indicators. We used a PCoA (which has as many principal coordinates as dimensions in the resemble matrix on which the eigen analysis is applied), of which only the first two coordinates (dimensions/axes) were shown. The percentage of the total variation contained in each of the two first principal coordinate is described in the text and is also provided in each PCoA (see Figure 3), and the scores in this case are the actual projections of the data points on the new 2D configuration map. The underlying ANOVA models in this case were a 3 (time) x 2 (salinity challenge) two-way ANOVA with a hanging control. The response (dependent) variables in this case are the 6 biochemical indicators (CAT, SOD, GST,

GSH, LPO, PO) and the explanatory (independent) variables are factor time (four levels) and salinity (two levels). We have made major changes to the text in order to clarify the points raised by Reviewer #2 and hope to have improved the fluency of the statistical methods we applied.

21) Fourth paragraph (lines 302-309): Is very confusing and in my opinion mistakes are present. It is not clear what are “the same statistical procedures”. Especially as in the previous paragraph you describe MANOVA, a multivariate model, but here LAC is a single response variable and cannot be analysed with a multivariate model.

Answer: We have modified the methods section and hope this is now clear.

22) The description of the analyses related to aerobic scope are completely lacking. Please add.

Answer: Thank you for bringing this up, we had included a phrase in the results section which has now been moved to the statistical analyses section.

RESULTS

1) General: keep in mind that your sample sizes per treatments are very low. (I understand that it is hard to obtain high numbers of these species.) Some non-significant results could be related to this. So, be reserved in your interpretations, and try not to over-interpret results. Use careful wording.

Answer: We have modified the text considering this suggestion.

2) You mostly show the transformed data. I think it would be valuable to report also absolute values of RMR and other response variables. This would be useful for comparisons with results of other similar studies, or even meta-analyses. If possible please add summary values in main text, and/or raw values in supplement as a data file. Sorry, if this was done, but it is not clear as you do not give a reference where raw data are deposited.

Answer: All RMR, BI and LAC data are available as a dataset file from the Zenodo repository (https://doi.org/10.5281/zenodo.8284963), which was also included in the original submission. However, we have added a supplementary figure for all BI showing the raw data of each enzyme, hoping that anyone could have a closer look. We have also included a reference to the raw data in the text (line 366), and after the acknowledgment section “data availability”.

Metabolic rates and behaviour during salinity challenges

3) Line 312: add “salinity”; use past tense, not present tense when describing results: were not significant.

Answer. Corrected.

4) Line 318: I see here that you did include the interaction of factors time and salinity in your models. But you did not say this in the methods. Please add.

Answer. We have included the description in the methods.

5) Lines: 319-321: These are the results of post-hoc pseudo-t tests. Please add the pseudo-t value along with the p-value.

Answer. The tables of all analyses have been modified and now include a letter showing significant differences after the post-hoc tests.

6) Line 331-332: Judging from the plot it is not obvious that RMR is higher in moderate than in extreme salinity; it seems similar. Please recheck the data, plot and stats for mistakes. Also, the interaction was not significant, so your statement “time has a

differential impact on the RMR regardless of the salinity challenge" is wrong. It would be true if the interaction would be significant.

Answer: Statistical comparisons of RMR between an abrupt change to moderate and extreme salinity were carried out using a two-way model, in which the effect was decomposed in two main terms and an interaction. If the interaction term resulted significant, it would mean that differences in RMR through time were different depending on the magnitude of salinity. This was only true for *T. mitchelli*. By contrast, in *T. dzilamensis* and *T. pearsei* differences through time (which in both cases were statistically significant) occurred in similar magnitude and direction regardless of whether individuals were challenged with a moderate or an extreme salinity. Only in *T. pearsei* were there mean significant differences in RMR (irrespective of time) between moderate and extreme salinity (as indicated by the main salinity term). We have added a letter to the plot indicating the significant differences. The description of results for T. pearsei has been re-written for clarification, following this suggestion (lines 389-390).

7) Lines: 332-335: Here you describe the results of post-hoc test, but it is very hard to understand, and it is not done in the same way as for the previous species (stats are not included). I suggest you gather all post-hoc test results in a table. Then you can exclude the boring stats from the text and just summarize the main results/pattern. This way would be much more useful and improve clarity. On the other hand, current Table 1 in mostly redundant and can be deleted; see my comment below.

Answer: We have extensively modified the results in order to make this section more articulate and relate the measured RMR values together with the results of the statistical tests and the behavioural observations of the *Typhlatya*.

8) Table 1: There is no need to include the whole table; there is too much information that are not very useful for the readers. I would report only the pseudo-F and p-values. You can do this in text, there is no need for a table. For example: "RMR in native salinity conditions were not significantly different among species (pseudo-F: 0.09, p-value: 0.931)." I would advise to do similar for per species results (report in text model results for both factors' main effects and their interaction). Minor: explanation of the table content is usually given above, not below the table. Below we only give minor annotations.

Answer. We have modified tables and now include the pseudo-F and p-values, along with results of post hoc tests where significant differences occurred.

9) Figure 2: I recommend marking the significant differences with asterisks or letter codes on plots for moderate and extreme salinity challenge.

Answer. We have added letters that identify each significant difference among groups, and converted the stress range bar into an arrow, in hope of giving the reader a sense of direction of the process. The figure description has been modified accordingly.

10) The last three paragraphs are confusing and need rewriting. You start with reports on behaviour, but it turns out you speak of RMR most of the time. This is confusing for the reader. I suggest doing the following: 1) keep only stuff related to behaviour and mortality and organize it into one paragraph; let it be the first one in this results section. 2) reuse the remaining text (lines 345-348, 352-356, and 360-364) and meaningfully include it in the description of RMR results.

Answer. We have re-written the paragraphs as suggested.

Antioxydant system

11) It is hard to evaluate these results as the related statistical methods are confusingly and insufficiently described.

Answer. Statistical methods have been re-written and expanded in hope of a clear and straightforward description.

12) Line: 385: statistically significant differences.

Answer. Phrase has been modified.

13) Line 388: statistically similar is an inappropriate term. Please use not statistically significantly different instead.

Answer. Phrase now reads "… *T. pearsei* was not significantly different from the other two species."

14) Line 392: replace sample by individuals or species. (2x).

Answer. Replaced

15) Line 395: delete vertical.

Answer. Deleted

16) Table 2: same comment and suggestion as for current Table 1.
    Answer. We have modified all tables.

17) Line 417: until here T. dzilamensis was always discussed after T. pearsei. Please keep this order throughout the manuscript.

Answer. Order in text and figure 3 have been modified accordingly.

Lactate

18) It is hard to evaluate these results as the related statistical methods are confusingly and insufficiently described.

Answer. Methods section has been adjusted and is now hopefully clear.

19) Line 461: "in their" seems out of place. Please check.

Answer. This phrase has been checked and amended.

20) Table 3: same comment and suggestion as for current Table 1 and 2 and comment that relate to that. Currently, reposting of results especially stats in this section is not clear and appropriate. Applying my suggestion will solve this issue.

Answer. We hope the new display of results in tables is succinct and makes the analyses clear.

21) Figure 4: replace "behavior" in the title with a more appropriate word like "content"

Answer. Replaced, thanks for suggesting that.

Aerobic scope

22) It is hard to evaluate these results as the related statistical methods are not provided in the methods section.

Authors. A new paragraph has been added to the section for statistical analysis regarding the aerobic scope.

DISCUSSION

1) Figure 6: I like this graphic summary of your results very much!

Answer. Thank you! We feel this captures the essence of this work =)

2) Line 615: "which can" is out of place. Either a sentence is lacking, or you should delete these words.

Answer. It has been deleted.

3) Line 609-630: Nice point! I agree the dynamics of haloclines in the environment are an important selective factor. I am wandering if you have seasonal data regarding this for the cenotes where these shrimps live and where you sampled. Is the halocline at Tm location more stabile than at locations of the other two species? Can you add a few short sentences regarding this to this paragraph?

Answer. We do not have seasonal data; however, we have referenced studies that have addressed these dynamics in similar anchialine sites in the Yucatan. We have added a paragraph into the discussion that expands on this point. The Tm population from As Tp were sampled from an inland cenote, they do not have access to a deeper MG layer. It is most likely, that these individuals have never been in a different salinity, but other populations and their ancestors have.

4) Your results implicitly suggest that colonisation of freshwater by marine species is easier than vice versa. This is interesting from an evolutionary standpoint. Does it match with existing knowledge, literature? Can you add a short discussion on this?

Answer. We have added a paragraph highlighting the phylogeny of the Yucatan *Typhlatya*, noting that their last common ancestor inhabited freshwater, and that *T. dzilamensis* is a diversion from the ancestral habitat. We discuss that this trait could potentially imply that *T. dzilamensis* has retained physiological traits that enable it to "return" to the FG environment.

5) At the end of the discussion clearly connect back to the introduction, its main story on climate change impact in caves.

Answer. See next answer.

CONCLUSIONS

1) I think this chapter is not really needed. Here you are repeating what you said already a few times in the manuscript and at this point it sounds boring already. I suggest to drop out this section and include its novel parts (Tm being at greater risk due to rising sea levels) into the discussion.

Answer. After consideration, we insist on keeping this section as the summary of our findings. However, we have tried to minimize repetition and have incorporated the risk of T. mitchelli into the last paragraph, also connecting with the introduction.
